# Lectins: Getting Familiar with Translators of the Sugar Code

**DOI:** 10.3390/molecules20021788

**Published:** 2015-01-22

**Authors:** Sabine André, Herbert Kaltner, Joachim C. Manning, Paul V. Murphy, Hans-Joachim Gabius

**Affiliations:** 1 Institute of Physiological Chemistry, Faculty of Veterinary Medicine, Ludwig-Maximilians-University, Veterinärstr. 13, 80539 Munich, Germany; E-Mails: Sabine.Andre@lmu.de (S.A.); kaltner@lmu.de (H.K.); j.manning@lmu.de (J.C.M.); 2 School of Chemistry, National University of Ireland Galway, University Road, Galway, Ireland; E-Mail: paul.v.murphy@nuigalway.ie

**Keywords:** adhesion, ganglioside, glycocluster, glycosylation, kidney, sialylation

## Abstract

The view on the significance of the presence of glycans in glycoconjugates is undergoing a paradigmatic change. Initially mostly considered to be rather inert and passive, the concept of the sugar code identifies glycans as highly versatile platform to store information. Their chemical properties endow carbohydrates to form oligomers with unsurpassed structural variability. Owing to their capacity to engage in hydrogen (and coordination) bonding and C-H/π-interactions these “code words” can be “read” (in Latin, *legere*) by specific receptors. A distinct class of carbohydrate-binding proteins are the lectins. More than a dozen protein folds have developed carbohydrate-binding capacity in vertebrates. Taking galectins as an example, distinct expression patterns are traced. The availability of labeled endogenous lectins facilitates monitoring of tissue reactivity, extending the scope of lectin histochemistry beyond that which traditionally involved plant lectins. Presentation of glycan and its cognate lectin can be orchestrated, making a glycan-based effector pathway in growth control of tumor and activated T cells possible. In order to unravel the structural basis of lectin specificity for particular glycoconjugates mimetics of branched glycans and programmable models of cell surfaces are being developed by strategic combination of lectin research with synthetic and supramolecular chemistry.

## 1. Why Glycans Are the Most Versatile Platform for Coding

**Figure 1:**
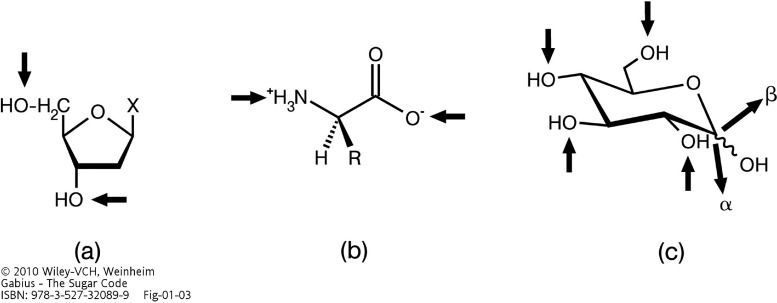
Illustration of the linkage points for oligomer formation in biomolecules by arrows. The phosphodiester bond in nucleic acid biosynthesis (**a**) and the peptide bond in protein production (**b**) yield linear oligomers. For each reaction, the same types of functional groups are involved throughout polymerization. In contrast, the glycosidic linkage in oligosaccharides can involve any hydroxyl group, opening the way to linear and also branched structures (**c**). Using d-glucose as an example, its active form UDP-Glc enables conjugation of this sugar to carbohydrate acceptors to any hydroxyl group of carbohydrate acceptors, as symbolized by arrows directed towards the hydroxyl groups. The anomeric position in chain elongation can be α or β, as symbolized by two bold arrows pointing away from the molecule (from [1], with permission).

Biochemical coding can be likened to a language. Oligo- and polymers are the biochemical equivalents of “words” and “sentences”. Built from the alphabets of nucleotides and amino acids, the information content of nucleic acids and proteins is determined by the sequence (order of “letters”). In both cases, the “letters” are connected in the same way (type of linkage and points of attachment; Figure 1a,b). DNA replication and protein production are therefore possible with a minimum of enzymatic steps at the required high accuracy. Inevitably, limits to the coding capacity are set this way. It is only the order of building blocks (and no further variable) that defines the primary structure. In quantitative terms, permutations of building blocks are the sole factor, which generates sequence complexity. A certain limit of oligomer length must thus be surpassed to store information without ambiguities. Is a class of biomolecule available for high-density coding?

**Figure 2:**
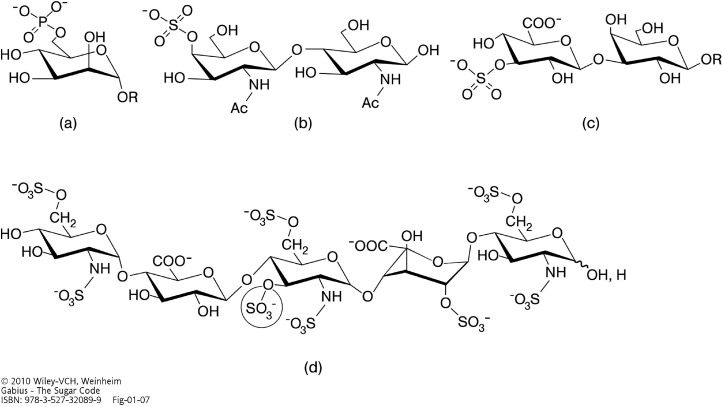
Illustration of phosphorylated (phosphated) and sulfated (sulfurylated) glycan “words”. 6-Phosphorylation of a mannose moiety (in the context of a pentamannoside) is the key section of a routing signal (“read” by P-type lectins) in lysosomal enzymes (**a**), 4-sulfation of the GalNAcβ1,4GlcNAc (LacdiNAc) epitope forms the “postal code” for clearance of pituitary glycoprotein hormones from circulation by a hepatic endothelial cell receptor, a bifunctional lectin with C-type and β-trefoil domains (**b**), the HNK (human natural killer)-1 epitope (CD57, 3-sulfated GlcAβ1,3Galβ1,4GlcNAc) is involved in cell adhesion/migration in the nervous system (**c**) and the encircled 3-O-sulfation in the pentasaccharide’s center is essential for heparin’s anticoagulant activity (**d**). All sugars are in their pyranose form. Please note that the central glucosamine unit has N,O-trisulfation and that the 2-sulfated iduronic acid (IdoA), given in the ^1^C_4_ conformation, can also adopt the hinge-like ^2^S_O_ skew-boat structure (about 60% or more for the ^2^S_O_ form in equilibrium depending on the structural context) when present within glycosaminoglycan chains of the proteoglycan heparin. 2-Sulfation of IdoA serves two purposes: favoring the hinge-like ^2^S_O_ conformation and precluding reconversion to glucuronic acid (from [1], with permission).

A brief look at carbohydrate biochemistry reveals the much larger potential of the sugar alphabet for coding: the sugar ring presents several chemically (nearly) equivalent hydroxyl groups. Each one (in the pyran/furan ring or in exocyclic position) can in principle be engaged in a glycosidic linkage with the anomeric center of the second unit in a disaccharide (Figure 1c). Connections can thus be made with different linkage points, from position 1 to positions 1, 2, 3, 4, or 6. As Figure 1c also makes obvious, the anomeric center can be in α- or β-configuration. Variation in the involvement of hydroxyl groups in the glycosidic linkage, together with the parameter of anomery, as highlighted in this figure, increases the coding capacity far beyond simple sequence permutations [2]. Each monosaccharide of the sugar alphabet has its characteristic set of points enabling enzymatic elongation. Often, each theoretically possible site is indeed recruited in glycan synthesis, as outlined for the “letter” l-fucose in the next paragraph. Furthermore, introduction of substitutions by phosphorylation or sulfation extends the biochemical diversity of the sugar alphabet [1]. To illustrate these two structural aspects, *i.e.*, variations in the glycosidic linkage and presence of substitutions, examples for bioactive sugar “words” ranging from a substituted monosaccharide (*i.e.*, 6-phosphorylated mannose) to a pentasaccharide with several sulfate groups are given in Figure 2. In contrast to nucleic acid/protein biosynthesis a large number of enzymes for chain initiation/elongation and modification is necessary. Obviously, the enzymatic machinery (assembly line) is capable of realizing the structural potential of sugars [3,4,5]. Whatever the requirement, whether for example additions of fucose or sialic acid at terminal or branch positions or specific chain elongations, e.g., by β1,3-linkages, are concerned, a broad array of glycosyltransferases (in the order of about 200 genes) is encoded in the genome [6,7,8,9,10,11]. The same applies to chain-modifying enzymes, with 35 sulfotransferases known to process glycoconjugates in the Golgi region [12,13].

**Figure 3:**
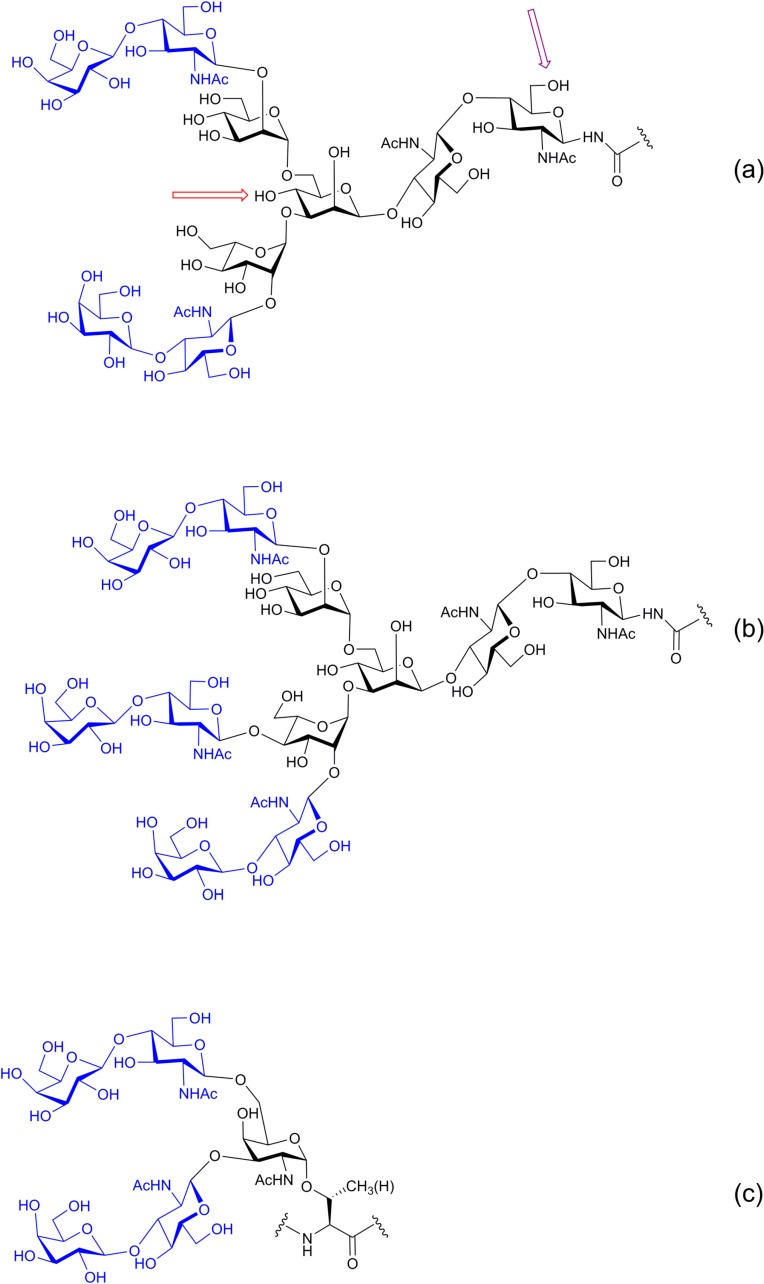
Schematic illustration of physiologically branched glycans. Complex-type N-glycans are constituted by the invariable core pentasaccharide (Man_3_GlcNAc_2_) and the peripheral region, here with extension of the two antennae (**a**) or introduction of a third branch to form a triantennary N-glycan (**b**). The sites for introduction of core substitutions at the innermost GlcNAc moiety (by α1,6-fucosylation) or the central Man residue (by β1,4-conjugation of the bisecting GlcNAc) are labeled by arrows (a). Core branching in mucin-type O-glycans is based on β1,3-/β1,6-extensions from the central GalNAc moiety (**c**). For structures and preparation of synthetic mimetics of the bi- to trivalent N/O-glycans, please see below (Scheme 1, Figure 12, Figure 13 and Figure 14 and Scheme 2).

In further contrast to nucleic acids and peptides/proteins, glycans are branched physiologically. Looking at standard protein glycosylation, complex-type bi- and triantennary N-glycans and a mucin-type core 4 O-glycan illustrate the capacity of monosaccharides to connect to three partners (Figure 3). At termini of glycan antennae, presence of branching is manifested by the epitopes of the histo-blood group ABH or Lewis systems, with fucose in α1,2-, α1,3- or α1,4-linkage [1,8,10]. Each linkage is formed by a distinct fucosyltransferase, the structurally related glycans have their particular “meaning”. As will be explained in the third section below, antibodies against blood group determinants were historically the first type of sugar receptors known to distinguish very similar “words”. Substantiating the versatility for isomer generation, even α1,6-fucosylation is possible. Not at termini but within the core, at the innermost N-acetylglucosamine (GlcNAc) moiety of N-glycans conjugated to an asparagine of the protein, an α1,6-fucosyltransferase can add the so-called core fucose (Figure 3a). Knock-out mice for fucosyltransferases and work with synthetic glycan chains without/with the substitutions have underscored that this non-random positioning indeed has its physiological significance [14,15,16]. Further looking at the core, a second type of substitution occurs in mammalian complex-type N-glycans: a GlcNAc moiety can be attached to the central mannose unit like a wedge in β1,4-linkage, referred to as the bisecting GlcNAc (Figure 3a). In the concept of the sugar code these intimately controlled core substitutions should have their own importance. Both the bisecting GlcNAc moiety and the core fucose unit are not inert additions to the N-glycan but act on its conformational behavior like molecular switches. They shift the conformational equilibrium and hereby alter the distances between terminal sugars, a key factor for affinity to receptors [17,18]. The realization of the role of branching for lectin reactivity has inspired synthetic work on glycoclusters, as further discussed in Section 6. The given examples are meant to convey the message that glycan assembly has reached a remarkable level of sophistication and exploits the theoretical possibilities of oligosaccharides for high-density coding, and this even with species-specific traits [19]. In conclusion, “carbohydrates form the third alphabet of life. Compared to amino acids and nucleotides their versatility for isomer formation (code words) is unsurpassed” [1].

This brief survey on glycan structures documents that simple sequence permutations are just one aspect of the primary structure. What is stated quite matter-of-factly has enormous consequences for experimental analysis. As explained above, the complete definition of any di- or oligosaccharide in structural terms requires the determination of the linkage points and the type of anomery. Addressing these issues makes carbohydrate sequencing (and synthesis) much more demanding than respective work on nucleic acids and proteins, explaining the origin of the often used term “complex carbohydrate”. As platform to store information, however, glycans offer the largest potential. Whether the words of the sugar language can then actually be read depends on the suitability of glycans to engage in interactions. This property must be assessed next.

## 2. Why Glycans are Suited for Biorecognition

Examining the structural details of glycans as presented in Figure 2 and Figure 3 has pinpointed a fair abundance of hydroxyl groups. They can readily be involved in hydrogen bonding or in coordination bonds with an appropriately positioned cation such as Ca^2+^. These interactions with a receptor site do not only provide enthalpic gains but also topological specificity by sensing complementarity. In addition, the set of remaining C-H bonds, slightly polarized, has a special ability. It shapes patches or contiguous regions for van der Waals interactions, especially with π-electron systems, then leading to C-H/π-interactions, with favorable thermodynamic consequences enthalpically and also entropically by reducing solvent accessibility [20,21]. Moreover, substitutions by negatively charged groups such as phosphate or sulfate, as shown in Figure 2, add contact points for ionic binding. Overall, these structural features set a strong basis for free energy generation upon binding.

When the glycan forms a complex with a partner, another factor must be considered: the binding process engenders changes in the spatial flexibility, e.g., by arresting a flexible molecule in a bound-state conformation. Automatically, this unfavorable entropy loss will need to be compensated. If intramolecular dynamics and structural fluctuations were limited, the extent of entropic penalty would be reduced, a reason to embed flexible peptide sequences into the context of proteins, that is to let them acquire a stable bioactive conformation. In the case of glycans, limiting the number of accessible conformers comes by their structural nature. The bulkiness of the pyranose rings imposes spatial constraints to free rotations around the glycosidic linkage. The conformational space becomes graded in terms of the energy, with “valleys” and “hills”, resulting in “topological” maps. As consequence, carbohydrates often access only a small set of energetically privileged conformers (“valleys” in the energy landscape) so that the sections of the energy “hills” are not populated [22,23,24]. Put metaphorically and with perspective, “the carbohydrate moves in solution through a bunch of shapes each of which may be selected by a receptor” [25]. Keeping in mind that synthesis of complex glycans depends on the binding and processing of carbohydrates by enzymes and that recognition of isomers by antibodies is a further physiological case of glycan binding, proteins are thus already known to be effective sugar receptors. Because a biomedical importance of glycans is assumed and their profile (glycome) is not only widely different on various cells, a challenge for analysis [26,27], but tightly controlled and regulated by synthesis and dynamic remodeling, especially by removing sialic acid from distinct chains with sialidases [28,29,30], one would intuitively expect an equally elaborated set of sugar receptors. All these factors, *i.e.*, suitability for hydrogen/coordination bonding and C-H/π-interactions, residence in energetically privileged structures, an elaborate synthetic machinery and ability of proteins to act as receptors, argue in favor of productive protein-glycan interactions.

Having up to this point focused on glycans, the question is whether a panel of sugar receptors matching the glycomic diversity has developed, beyond the glycan-processing enzymes and glycan-binding antibodies. This is indeed the case, on the level of protein folds and sugar specificities [31]; so the tools for reading sugar-encoded information (to read, *legere* in Latin) are available. They are called lectins.

## 3. Lectins: Definition and Overview

The term has its roots in work with plant proteins that have specificity for histo-blood group determinants. Classically, this property is measured by detecting agglutination of erythrocytes of donors with different ABH(0) status [32,33,34]. Origin and activity of the proteins are expressed in the term “phytohaemagglutinin”. This assay, the haemagglutination, had been instrumental to define blood groups, initially by using antiserum and the capacity of auto-antibodies to cross-link erythrocytes [35,36]. Actually, this reaction underlies fatalities in transfusion medicine. Thus, as noted above, antibodies in serum were known to distinguish between such clinically relevant surface epitopes, although their biochemical nature had at that time not yet been disclosed. This situation prompted W. C. Boyd to introduce a new term for blood-group-specific proteins from plants, which are not antibodies [37]. The name “lectin” is thus a means “to call attention to their specificity without begging the question as to their nature” [37], and, fittingly, a lectin (from eel serum) was crucial to reveal fucose as key determinant of the H(0) epitope [38,39]. Today, this term is used for (glyco)proteins with specificity for carbohydrate, different from sugar-specific antibodies and enzymes as well as from sensor/transport proteins for free mono- and oligosaccharides [40]. Non-catalytic domains associated with the enzymatic center of glycoside hydrolases, mostly in bacteria and fungi, are also kept separate, these protein parts referred to as carbohydrate-binding modules [41]. Because assays for lectin detection (for survey please see [42]) and powerful affinity chromatography (introduced 1965 [43]) paved the way for purification, it became possible to answer the question whether lectins are rare or frequent inventions during phylogenesis.

**Figure 4:**
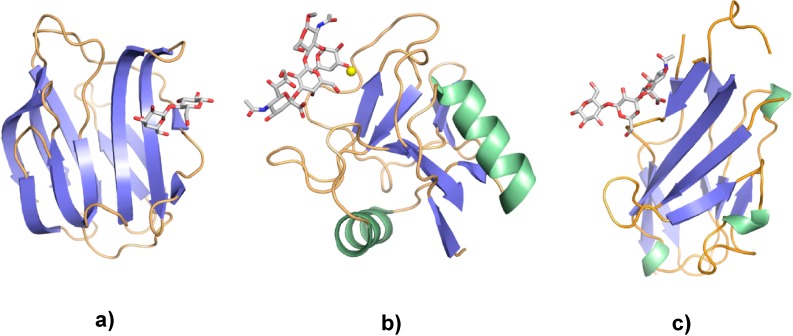
Illustration of three common lectin folds in complex with a ligand. (**a**) The β-sandwich of human galectin-1 in complex with the disaccharide lactose (PDB code 1GZW); (**b**) the C-type CRD of human E-selectin with its Ca^2+^ (yellow sphere) in complex with the tetrasaccharide sialyl Lewis^x^ (CD15s) (PDB code 1G1T); (**c**) the I(gG)-type CRD of the murine siglec sialoadhesin (siglec-1) in complex with the trisaccharide 3'-sialyllactose (PDB code 1QFO).

Crystallographic analysis of lectins is able to assign any protein to one of the many families based on the domain (fold) structure. Systematic classification has discovered more than a dozen folds in vertebrates with ability to accommodate a glycan [31,42]. A certain module can even be a scaffold for different groups of lectin, with the contact site for the ligand located in various regions of the common fold, as is the case for the β-sandwich [31,42,44]. Initially found in leguminous lectins (here concanavalin A [45]), this fold is characteristic for the pentraxins, galectins (please see Figure 4a and Section 4) and mannose-specific lectins in glycoprotein quality control and routing from the endoplasmatic reticulum to the Golgi [31,42,44]. Obviously, sugar recognition by lectins, in terms of number of folds and of fold usage, is not a singular event in Nature. Both glycan and lectin structures can reach equal levels of sophistication. Illustration of three examples of carbohydrate recognition domains (CRD) of mammalian lectins exemplifies the diversity of folds (Figure 4). Notably, the C-type fold (Figure 4b) harbors a Ca^2+^ in its center for topologically specific interplay with the ligand, a case of direct contact between the strategically positioned cation and the glycan, embedded in a network of hydrogen bonds between sugar and protein [41,46]. This orchestrated constellation, a sensor for complementarity, ensures monosaccharide specificity. Combined with the presentation of C-type lectin domains in a trimer, it explains the high avidity of binding of the hepatic lectin to the topologically complementary branch ends of the complex-type triantenary N-glycan, shown in Figure 3b [47,48,49]. Lectin sites thus cooperate and “read” both sugar structure and mode of presentation.

The sequencing of lectin genes disclosed a functionally salient structural aspect beyond the contact site to the glycan: the lectin domain can be associated with other types of modules to establish a mosaic of active sites [50,51]. Membrane-spanning sections, regions for forming α-coiled coils or (non-)triple helical collagen-like repeats for oligomerization, immunoglobulin-like (or sushi) repeats as spacers or as sites for recognition of other types of ligand, even other types of CRDs (a β-trefoil domain in the tandem-repeat-type (C-type) mannose receptor active in “reading” the “postal code” for delivery (clearance) of pituitary glycoprotein hormones (please see Figure 2b for epitope structure) or in lecticans binding hyaluronic acid), to list several examples, are encountered in this respect [52,53,54,55,56]. The structural organization in oligomers mentioned above is thus facilitated, as required for target specificity in the case of the just mentioned asialoglycoprotein receptor or of serum collectins [1,57,58,59]. In fact, there is a wide spectrum of functional cooperation between modules: different sites in a protein can interact with binding partners toward a certain end. In the case of lectin (discoidin I)-mediated ordered cell migration, sugar binding is recruited for lectin secretion from the slime mold cells, the lectin’s RGD site of extracellular lectin then for transient cell contacts with a cell surface receptor [60]. The large number of folds and the wide variety of structural contexts, in which CRDs occur, are strong arguments in favor of a broad physiological significance of lectins.

Having started their success story in hematology, lectins soon acquired the status of versatile reagents for glycan localization, isolation and characterization. Together with carbohydrate-specific (monoclonal) antibodies they have enabled a wealth of studies, collecting ample evidence for glycan presence and dynamics of regulation, spatially and structurally in cells and tissues. Carbohydrate specificity of a lectin was first detected in the case of concanavalin A by precipitation assays in 1936: “one part of glycogen in 500,000 parts of water will give a perceptible haziness if allowed to stand after the addition of excess of concanavalin A. In as much as this precipitation resembles the reaction between antigen and antibody, where the glycogen takes the part of the hapten, we were interested in investigating it.” [61]. The authors concluded that the hapten-like molecule, with which concanavalin A “unites, may be a carbohydrate group” [61]. Nowadays, a wide panel of methods will evaluate carbohydrate binding (for review, please see [42]), technically simply measured by high-throughput arrays [62,63]. These data sets are helpful to interpret results from the application of labeled lectins in routine cyto- and histochemistry, especially when regional or temporal regulation has been observed [64,65,66,67]. The bisecting GlcNAc residue, to epitomize the intimate connection, is readily localized by the bean lectin PHA-E (E: erythroagglutinin) [68]. That said, each lectin entering the list of reagents could open the door to new insights on specific epitopes so far not mapped. The broad screening for lectin activities in diverse plants, fungi and mushrooms thus has notable merit beyond its descriptive outcome, that it is to identify new tools with particularly useful features. The agglutinin from the mushroom *Polyporus squamosus* sets an excellent example in this respect.

This lectin is specific for the α2,6-sialyl branch end of N-glycans [69,70,71]. Sialylation can entirely change the bioprofile of a glycan branch, by reducing intermolecular interactions through charge repulsion passively or by switching off/on ligand avidity for lectins (such as galectins or siglecs) actively. Based on studies in cell biological models, in which expression of distinct genes such as tumor suppressors or oncogenes can be activated/silenced and the impact of the manipulation on glycosylation be defined, especially lectins with specificity to α2,3/6-sialylated epitopes (or to N-glycan core substitutions) have diagnostic value [72,73,74]. The detection of such changes then prompts to trace their physiological relevance. Toward this aim, the possibility of a productive interplay with an endogenous lectin constitutes an attractive hypothesis for further work. Whereas lectins from other (exogenous) sources substantiate presence of an epitope, lectin presence *in situ* could be the missing link between a glycan and a process. With hindsight somewhat surprisingly, it took more than two decades to bridge the gap from the rather popular phytohaemagglutinins (the synonym for plant lectins) to lectins in mammals, the large lectin contents in seeds (for concanavalin A about 2.1 g/100 g) probably being a factor. Pioneering studies on the role of sialic acid (presence/absence) in serum clearance of the glycoprotein ceruloplasmin led to the purification of the first mammalian lectin (from rabbit liver) [75,76,77]. Explicitly, this work proved that a glycan “word” can be “read” by an endogenous lectin, like a postal code by a delivery service, putting endogenous sugar receptors on the research agenda. Considering the diversity of glycan structure and the dynamics of changes, postulating broad functional facets of lectin-glycan recognition *in situ* is indeed an appealing concept that commands attention. Perspectives for applications first led to establish a solid foundation by searching for sugar receptors, then studying in detail each detected lectin from its gene and protein structures to the expression profile, finally the interaction with counterreceptors and its physiological consequences.

**Figure 5:**
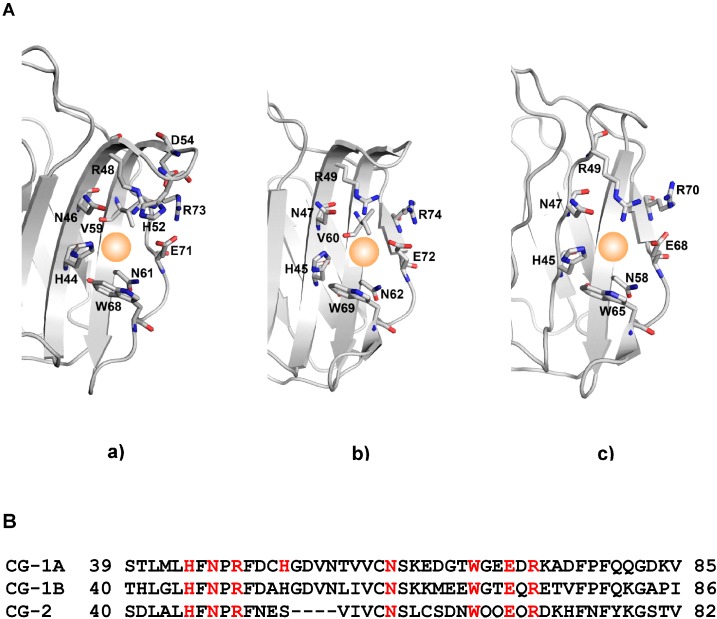
Comparison of the carbohydrate-binding sites (**A**) of the proto-type chicken galectins CG-1A (**a**, PDB code 1QMJ), CG-1B (**b**, PDB code 3DUI) and CG-2 (**c**, PDB code 2JMZ). The sequences (**B**) provide information on deviations in the vicinity of the conserved residues (in red), which contact the ligand.

Ideally, all members of a lectin family are analyzed together comprehensively in direct comparison. In principle, even two related proteins can have diverged to a level that their activity profiles will not or only to a small extent overlap. As domains in a multimodular protein do, individual family members may also cooperate in a network. What sounds rather academic and complex can best be illustrated by looking at the research strategy and emerging results in the case of a distinct family, preferably with biomedical relevance. In view of their involvement in diverse processes such as cell adhesion, growth regulation, mediator release and migration as well as intracellular activities such as regulation of gene expression or apoptosis [31,41,51,52,78,79,80,81,82], galectins serve as such an instructive test study object. They are lectins with specificity to a β-galactoside core, a β-sandwich fold (shown in Figure 4a) and a conserved sequence signature with a central Trp moiety for ligand contact via C-H/π-interactions [83]. The set of conserved amino acids is shown in Figure 5, as illustration of the carbohydrate-binding site and as sequence for three closely related homodimeric avian lectins. When inspecting Figure 5, the crucial Trp residue is easily spotted in the carbohydrate-binding sites of the three galectins.

The first member of this family was isolated from the electric organ of *Electrophorus electricus* in 1975 [84]. In this case, the classical haemagglutination assay and the inhibition of activity by lactose gave direction to purification, which was greatly favored by the presence of a reductive agent (β-mercaptoethanol) to protect this lectin from oxidation [83]. Ensuing work, first on the level of proteins, later of genes, has thoroughly mapped galectin presence in vertebrates and defined the intrafamily diversity, especially due to the growing number of sequenced genomes. Database mining, an approach to search for genes satisfying the criteria for homology and also for gene duplications, as successfully done for mammalian galectin-7 (Gal-7) [85], has substituted work in the cold room and peptide fingerprinting to draw a genealogical tree for the protein family. Doing so for galectins led to three branches.

## 4. Galectins: A Model to Trace Lectin Divergence

**Figure 6:**
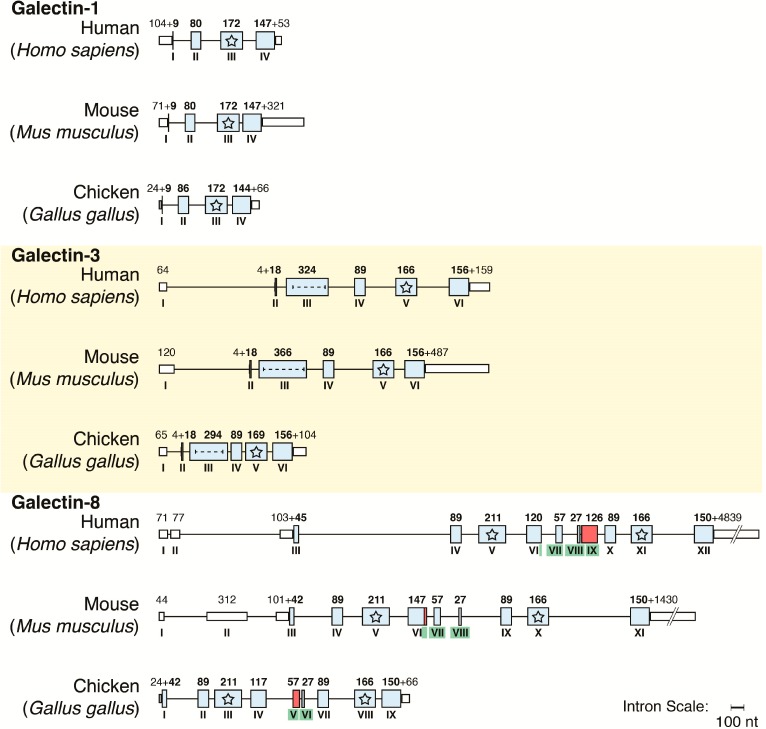
Phylogenetic conservation of galectin genes in vertebrates. For comparison, the structural organization of a gene from each group (Galectin-1: a proto-type protein with one CRD; Galectin-3: a chimera-type protein with one CRD attached to an N-terminal tail with a peptide and non-triple helical collagen-like repeats; Gal-8: a tandem-repeat-type protein with two different CRDs covalently connected by a linker peptide, present in two lengths due to alternative splicing) is presented, and this for man, mouse and chicken. Exons are given as boxes (colored when coding, further marked with a star when coding for the carbohydrate-binding sites), introns and 5'/3'-untranslated sequences as lines (lengths drawn in proportion). Arabic numbers provide sequence lengths, Roman numbers order of exons. The dashed line in exon III of the Galectin-3 gene depicts the repeat section, the green coloring of Roman exon numbers in the gene for Galectin-8 the coding for the linker peptide (34/76 amino acids in man, 33/42 amino acids in mouse, nine/28 in chicken).

Assignment of a galectin to each of the three categories is done by the structural mode of presenting the CRD. Two nomenclature systems are in use. They separate the proto-type (homodimeric), the tandem-repeat-type (two different CRDs covalently connected by a linker) and the chimera-type form, in which the CRD is associated with a tail (non-triple helical collagen repeats and a peptide with two sites for serine phosphorylation) [31,51,52,86]. Remarkably, occurrence of diversification is confined to the first two groups, whereas galectin-3 (Gal-3) is the sole chimera-type protein. On the level of the genes, galectins from the three groups in diverse organisms share a common exon-intron architecture. This conservation is illustrated exemplarily in Figure 6. These obvious traits of phylogenetic relationship, documented for avian and mammalian genes in Figure 6, have important implications for the selection of the model organism for studies aimed at delineating consequences of the intrafamily divergence. In other words, for a comprehensive study, a species with minimal number of galectin genes covering all three groups should best be suited for detailed characterization of structural aspects, binding properties and expression. With a total of five canonical proteins, the chicken galectins (CGs) fulfill this criterion [52,87,88]. Reflecting the high degree of homology to mammalian orthologues, they are referred to as CG-1A/B and CG-2 (proto-type), CG-3 (chimera-type) and CG-8 (tandem-repeat-type).

Moving from gene to protein sequence and then to folding [89,90,91], a common theme emerging from the comparison of crystal structures of the three proto-type CGs is the occurrence of sequence deviations in the vicinity of the strictly conserved residues (Figure 5). The architecture of the carbohydrate-binding sites given in panel (A) and the amino acid sequences to peruse substitutions (B) document both the homology and the divergence (Figure 5). As test case, the paralogues CG-1A/B, arisen from a gene duplication after separation of lineages of birds and mammals [92], are readily distinguishable (Figure 5). The changes in their sequences intimate consequences for the interaction with ligands. Assays with free glycans and glycoproteins revealed differences in binding to histo-blood group AB determinants and the type I N-acetyllactosamine (Galβ1,3GlcNAc) core between CG-1A/B [93,94]. Of note, exclusively CG-1B reacts to lactose binding in solution with a structural compaction measured as increase (by 5.6%) of the diffusion constant [95]. In addition to structural aspects of the protein, mapping regulatory gene sequences comparatively is equally important. Set in relation to the coding sequences of these galectin genes, the extent of deviations is relatively higher in the promoter regions [96,97,98]. The fact that presence of sequence motifs for binding transcription factors is subject to marked changes points to individually distinct expression profiles.

**Figure 7:**
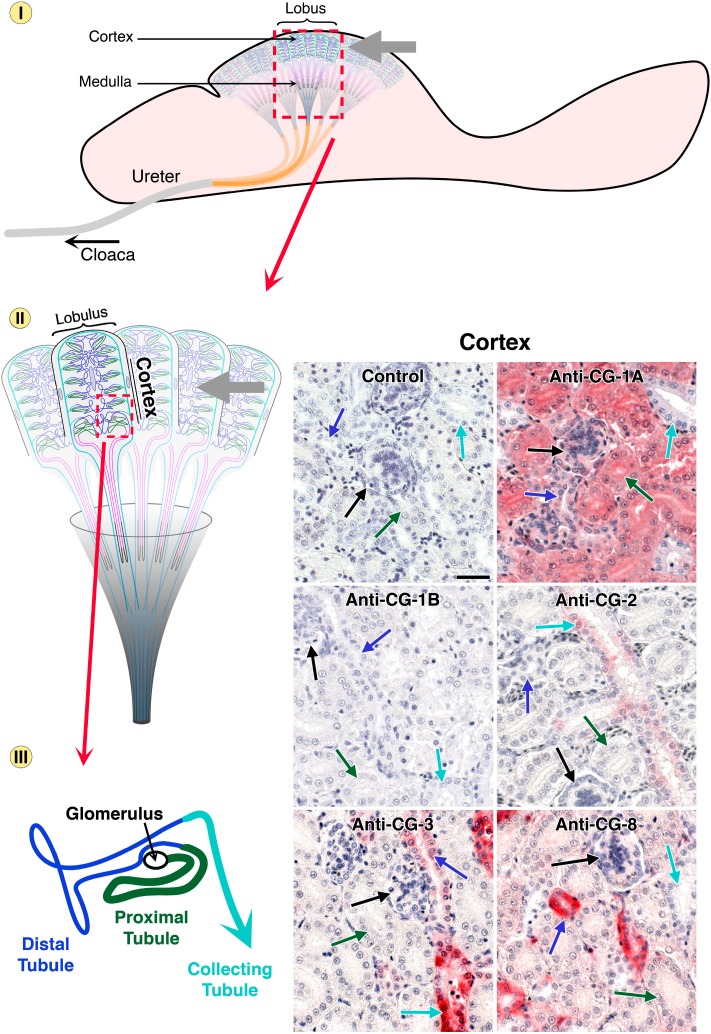
Schematic illustration of the chicken urinary system and its distinct regions on three levels of microscopical magnification. Starting with the lobus (dashed square, top, with the cortex region highlighted by the grey arrow), uriniferous tubules of the lobules (dashed square, middle) in the kidney cortex are organized in consecutive segments, given in different colors (bottom). Arrows in respective color point to these regions in the microphotographs of immunohistochemical galectin localization (bar = 25 µm). Control section was processed to reveal any antigen-independent staining (no incubation step with antibody in otherwise identical processing). Comparison of staining profiles discloses a qualitative difference in expression of the proteins of the paralogue pair CG-1A/B. On the cellular level, CG-2 and CG-3 were seen in cytoplasm and nuclei, CG-1A and CG-8 were confined to the cytoplasm.

**Figure 8:**
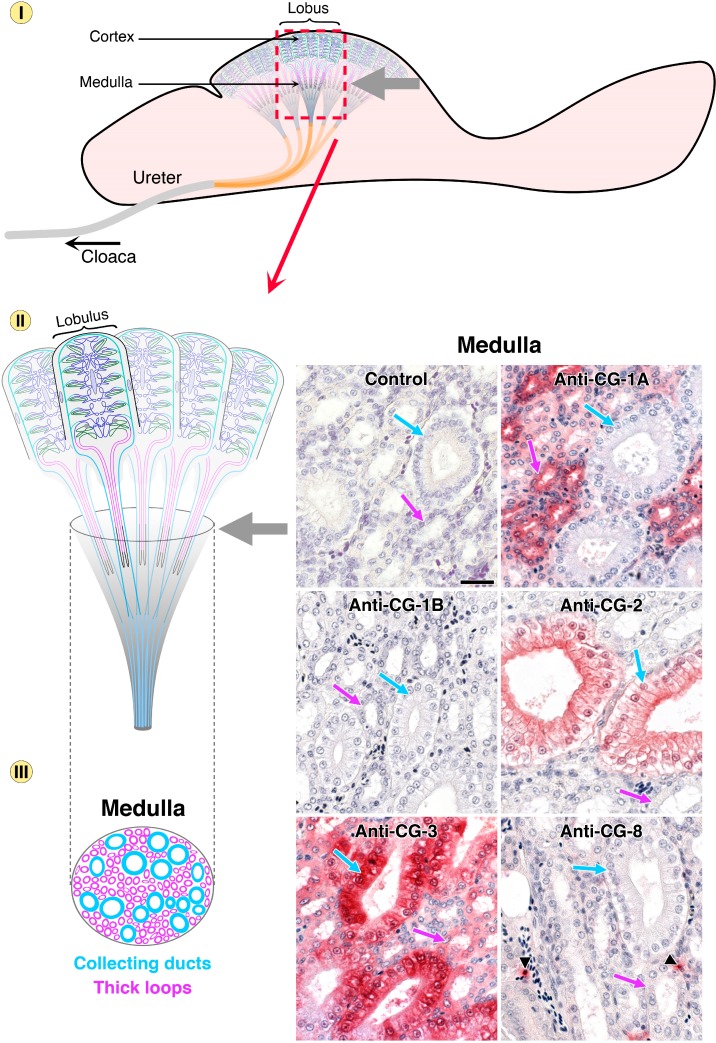
Schematic illustration of the chicken urinary system with focus on the medullary part of the kidney (top, grey arrow) with its collecting ducts and thick loops. Arrows in respective color point to these regions in the microphotographs (bar = 25 µm). Segmental specificity was revealed for CG-1A in epithelial lining of thick loops and CG-2 in collecting ducts, whereas no CG-1B could be detected and CG-8 positivity was exclusively seen in the connective tissue.

**Figure 9:**
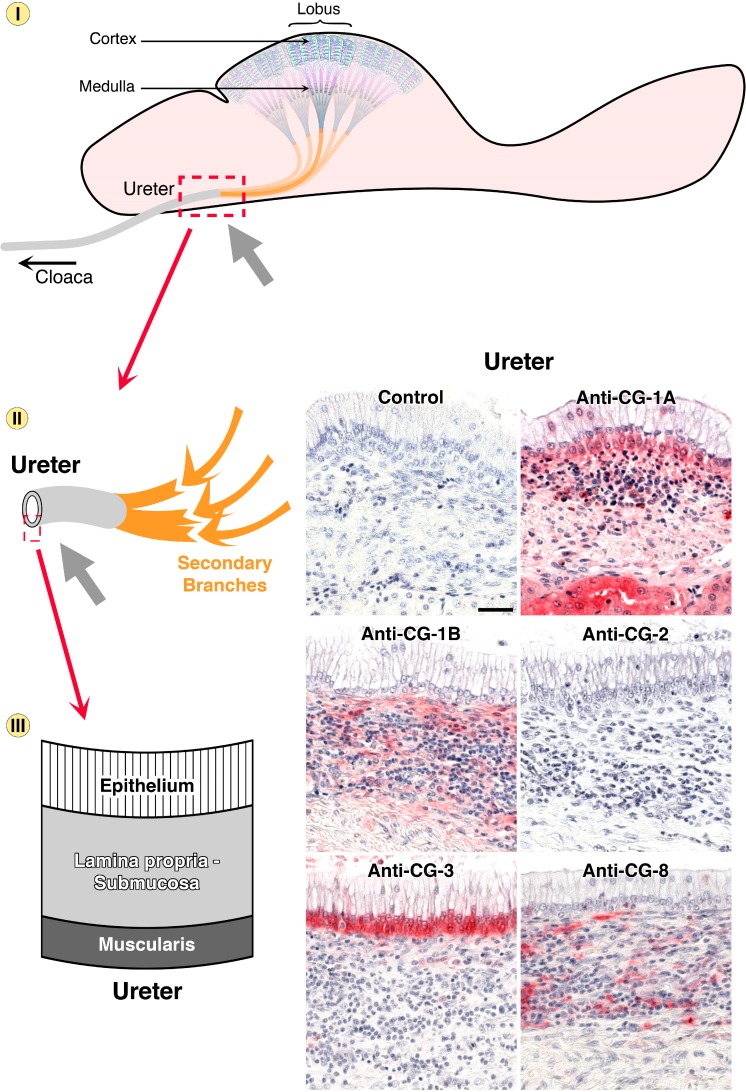
Schematic illustration of the chicken urinary system with focus on the ureter and its branches (top, grey arrow). Urine from secondary branches reaches the ureter constituted histomorphologically by the three parts given (left, bottom). CG-3 positivity was confined to the infranuclear portion of epithelial cells, CG-1B/CG-8 to connective tissue, while CG-1A was detected in all three layers (bar = 25 µm).

This is indeed the case. Using galectin-type specific antibodies, protein presence and localization can be determined in tissue sections (for detailed description of the quality controls for the antibody preparations and technical details of the immunohistochemical processing, please see [96,97,98]). Thus, the resulting microphotographs will answer the question on similarities and differences, especially if consecutive sections are stained and data presented for comparison. As graphic example, the series of Figure 7, Figure 8 and Figure 9 presents immunohistochemical detection of the five CGs in chicken kidney and ureter (together with the negative control to exclude any antibody-independent staining).

In the different parts of the avian urinary system, that is cortex (Figure 7), the medullary region (Figure 8) and finally ureter (Figure 9), qualitative differences and cell-type-specific positivity are seen. To exclude a false-negative result it is instructive to note that, by monitoring profiles in other regions and organs with these antibody preparations, CG-1B is detectable in respiratory epithelium or stratum intermedium of the epidermis, CG-2 in epithelial lining of the gut, along with CG-3 [88,96,98]. Measuring responsiveness to inducers of differentiation, also under the control of promoter sequence elements (for murine Gal-1 induction by butyrate, it is the 5' proximal Sp1 site at −57 [99]), extended the evidence for individual expression patterns, both for CG-1A/B and for human galectins, indicating similar cross-species reactivity [100,101]. Because a conserved gene architecture for murine genes was depicted in Figure 6, it is fitting to add that an immunohistochemical study on six galectins (−1, −2, −3, −4/6 and −7) in the digestive tract of mouse confirmed the conclusion on a network, on the levels of mRNA and of protein [102,103].

In summary, the sequence divergence (in coding and promoter sections) establishes a team of proteins with overlapping specificities for β-galactosides and individual expression profiles. In contrast to an expectation for rather similar (redundant) expression profiles of these homologous proteins, up to qualitative differences are observed in immunohistochemical monitoring of the five CGs, a result obtained for several vertebrate species. With medical relevance, studies on human tumors with more than one antibody further validate this network concept (for recent review, please see [104]). Galectins, in the avian model and in mammals, appear to be suited for distinct functions in a tissue- or cell-specific context, and this message can be extrapolated to other classes of lectins like C-type lectins and siglecs (for illustration of these folds, please see Figure 4b,c; for introductions to structural divergence in these lectin classes, please see [53,55]). However, that does not necessarily mean that there are no alterations between lineages and species. For galectins, the presence of genes can be species- or even strain-specific, in the cases of rat galectin-5 and mouse galectin-6, respectively [105,106,107,108]. The size of the CD33 group of siglec genes is markedly different between rodents and man, the consequence of a loss in respective coding sequences [109,110]. By the way, siglec binding to glycans requires presence of a sialic acid. Its detection in a distinct linkage type, for example in α2,6-linkage, is therefore possible not only by fungal, plant or slug lectins, as mentioned above for the *Polyporus* lectin, but also by human siglecs such as CD22 [111]. Connecting to the application of plant lectins for glycan detection, localization (glycophenotyping) and characterization, it is obvious that this approach using endogenous receptors will go beyond the descriptive level.

## 5. Endogenous Lectins as Tools and Effectors

**Figure 10:**
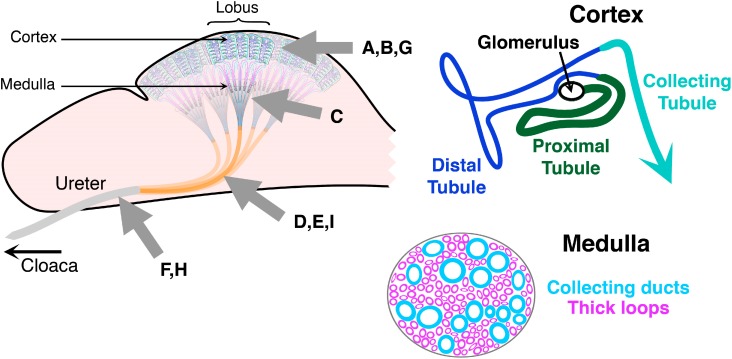

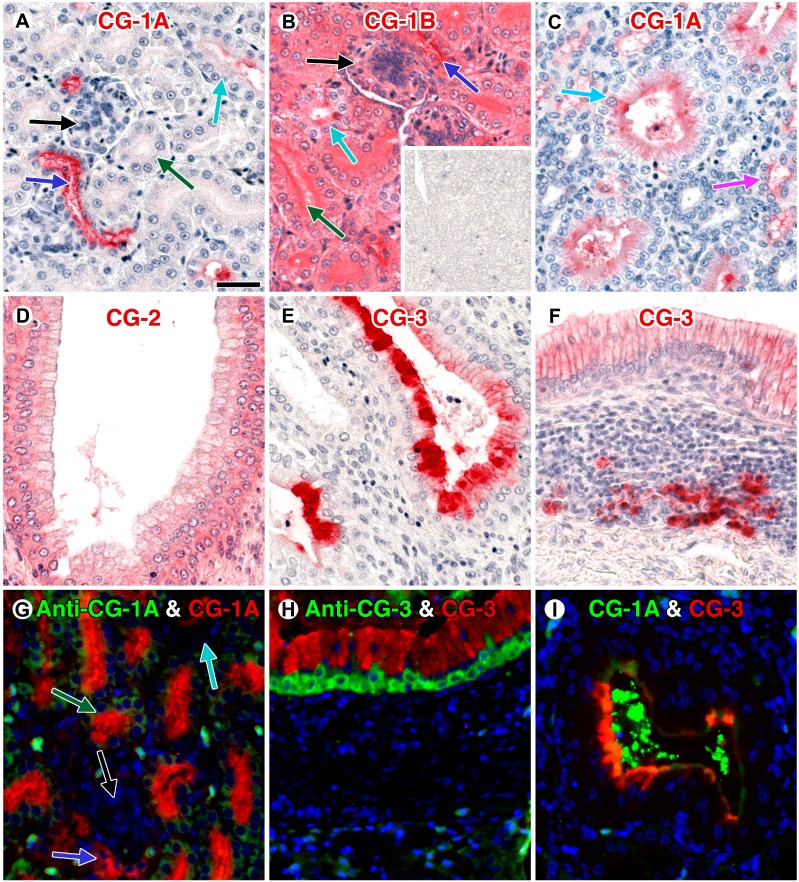
Galectin histochemical detection of accessible binding sites for tissue lectins in the kidney cortex (**A**,**B**), medulla (**C**), secondary branches of the ureter (**D**,**E**) and the ureter (**F**). The color coding of the arrows follows the segmental coloring for cortex and medulla (top, right). An examplary control for inhibition by the cognate sugar is shown as inset in panel B. Very distinct staining is detected on apical surfaces in the epithelial lining of renal tubule segments in the cortex for CG-1A and for CG‑3 in the apical region of secondary branches of the ureter (E). Co-localization of galectin and accessible binding sites by fluorescence microscopy in cortex (**G**) and ureter (**H**) revealed availability of reactive sites. Sequential application of two fluorescent CGs disclosed regional specificity of binding sites for CG-1A in the ureter (**I**). Mostly lumen and small areas of the epithelium (CG-1A) and apical surface of the epithelium (CG-3) were positive. For technical details of galectin labeling and of the galectin histochemical staining protocol, please see [111,112,113]. Bar = 25 µm.

As done with plant lectins, a tissue receptor can be labeled under activity-preserving conditions to become a laboratory tool. Lectin histochemistry with endogenous receptors determines tissue reactivity for each type of tested protein, and the question is whether similar proteins will differ in binding to tissue sections. As exemplarily illustrated in Figure 10, staining patterns can be non-uniform in a section, confined to a region, and indeed different in comparison between galectins. Combined with immunohistochemistry, localization of lectin and of sites accessible to a lectin can be visualized in the same section (Figure 10G–I). These binding features can then be correlated to biological processes or to the clinical course of a disease such as tissue fibrosis or malignancy. Assessing binding capacity with a galectin in human tumors, Gal-3 has been documented to be of prognostic relevance in two types of carcinoma [112,113]. To biochemically identify and characterize endogenous counterreceptors, affinity chromatography and co-immunoprecipitation of lectin-glycoconjugate complexes coupled with mass spectrometric identification of the binding partners have proven instrumental. Respective work identified laminin and carcinoembryonic antigen as counterreceptors in human colon cancer cells [114] (please see also below for counterreceptors active in negative growth regulation). Of note, work on mammalian lectins in general disclosed that they bind to rather few cellular glycoconjugates, therefore termed counterreceptors. The reasons for restrictions on epitope presence on glycoproteins and for homing in on only few targets are not yet fully understood, an incentive for research. Of practical value, tissue lectins can be used as tools in an as versatile manner as plant lectins are, with the perspective for delineating physiological significance.

**Figure 11:**
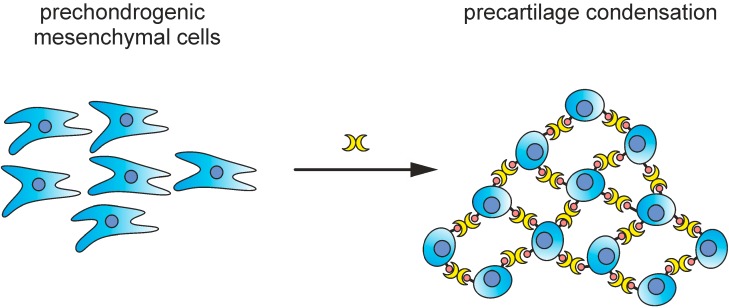
Illustration of the assumed role of homodimeric CG-1A to bridge precartilage mesenchymal cells. This CG targets specific glycoconjugates, which can be considered as functional biomarkers for prospective sites, where condensations will be formed [115]. In addition to cell aggregation (*trans*-interaction) crosslinking of cell surface glycoconjugates (*cis*) can trigger outside-in signaling to promote differentiation and proliferation. Of note, human cartilage expresses several galectins, with correlation to the state of degeneration in osteoarthritis [116].

Turning to a CG in functional terms, a role in avian limb skeletal morphogenesis has been inferred both by its detection at prospective sites of condensations in the embryo and by formation of regular arrangements of the mesenchymal cells by the homodimeric CG-1A [115]. The underlying principle of the cross-linking of cells by the lectin is illustrated schematically in Figure 11. Working as effector, lectin-dependent aggregation of glycoconjugates (lattice formation) on the surface is then followed by outside-in signaling, leading to differentiation [115]. Shifting the balance between proliferation and anoikis/apoptosis, inducing mediator release, stimulating cell migration and invasive properties and protecting the host by defense reactions based on recognizing microbial surface epitopes belong to the activity spectrum of galectins (for review, please see [41]). Selected examples presented in the next paragraph illustrate the intimate interplay with regulatory events on glycan synthesis for effector activity.

Activity of a lectin strictly depends on the presence of the cognate counterreceptor(s). As noted above, despite the abundance of glycans on the cell surface, tissue lectins have the ability to sense structural and topological factors for this productive interplay. They are grouped into six categories: the specificity to mono- or disaccharides (level 1) and to oligosaccharides (level 2), the selection of oligosaccharide shape (conformer) from an equilibrium (level 3), the preference to particular spatial presentations of glycan chains in a glycoprotein with respect to orientation of branches and to clusters of headgroups in bi- to pentaantennary glycans (level 4) and to clusters of headgroups of neighboring chains in a glycoconjugate (level 5), finally the preference to glycan presentation in microdomains or similar aggregates (level 6) [41]. In the case of galectin-1 (Gal-1) and induction of apoptosis, the glycan counterreceptor is the mucin-type core 2 O-glycan structure on prostate (LNCaP) cells [117], on activated effector T cells it is the pentasaccharide of ganglioside GM1 [118]. In both cases, clustering of cognate headgroups (covalently by branching as seen in Figure 3c or non-covalently in microdomains) is most likely a discriminatory feature. In fact, harming microdomain integrity by extracting cholesterol from neuroblastoma cell membranes caused an about 10fold decrease in affinity for Gal-1 [119].

Remarkably, the lectin, depending on the cellular context, “reads” different glycans, presented by a glycoprotein as N- or O-glycosylation and a glycolipid such as GM1. On the glycoprotein level, CD7, CD43, CD45 or an integrin are the functional counterreceptors to start Gal-1-triggered signaling toward T cell apoptosis/carcinoma growth arrest [120,121,122,123,124,125,126]. Of note, the homodimeric Gals-2 and -7 use separate effector pathways in the caspase cascade toward the same end, that is programmed cell death of activated T cells [127]. *In situ*, both lectin and glycan expression can be intimately co-regulated toward an optimal effect. Here, master regulators appear active, as delineated for anoikis induction in pancreatic cancer cells (Capan-1) *in vitro* by tumor suppressor p16^INK4a^ via upregulation of Gal-1 (the lectin) and the fibronectin receptor (also termed α_5_β_1_-integrin, the protein scaffold) as well as the downregulation of sialic acid biosynthesis to increase responsiveness to Gal-1 by reducing α2,6-sialylation, a block to Gal-1 binding [73,128]. At the same time, Gal-3 is downregulated, because it would competitively block Gal-1 binding without inducing pro-anoikis signaling via the α_5_β_1_-integrin [129]. The difference in CRD presentation (homodimer *vs*. monomeric CRD with tail) underlies this functional disparity, a case of functional antagonism between galectins. Obviously, the way of cross-linking within the resulting complexes on the cell surface is crucial for triggering the response. Thus, in addition to the glycan sequence, local density by clustering in a glycoprotein (level 4), as shown in Figure 3, can be a factor to predestine sites for lectin binding. This assumption must be verified experimentally, a difficult task on the level of cells, where engineering with surgical precision is hardly possible. To start with simple systems and then to increase the complexity stepwisely in model studies for delineating respective structure-activity relationships is possible for synthetic and supramolecular chemistry. The first step into this direction is to prepare a synthetic core presenting several glycans (glycocluster).

## 6. From Glycoclusters to Artificial Cell Surfaces

**Scheme 1:**
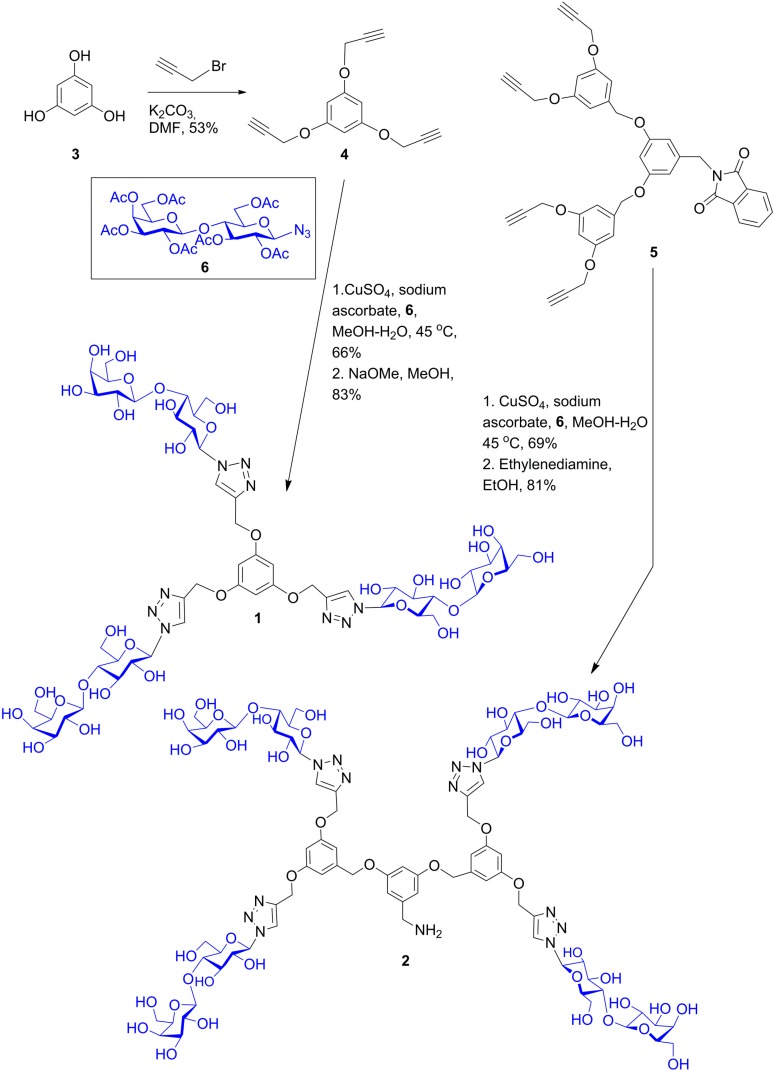
Synthetic routes to the trivalent (**1**) and tetravalent (**2**) glycoclusters via copper-promoted azide-alkyne cycloaddition as central step using 1,3,5-tris(alkynyloxy)benzene (**4**) and 2-(3,5-bis(3,5-bis(prop-2-ynyloxy)benzyloxy)benzyl)isoindoline-1,3-dione (**5**) (for experimental details, please see [131]).

**Figure 12:**
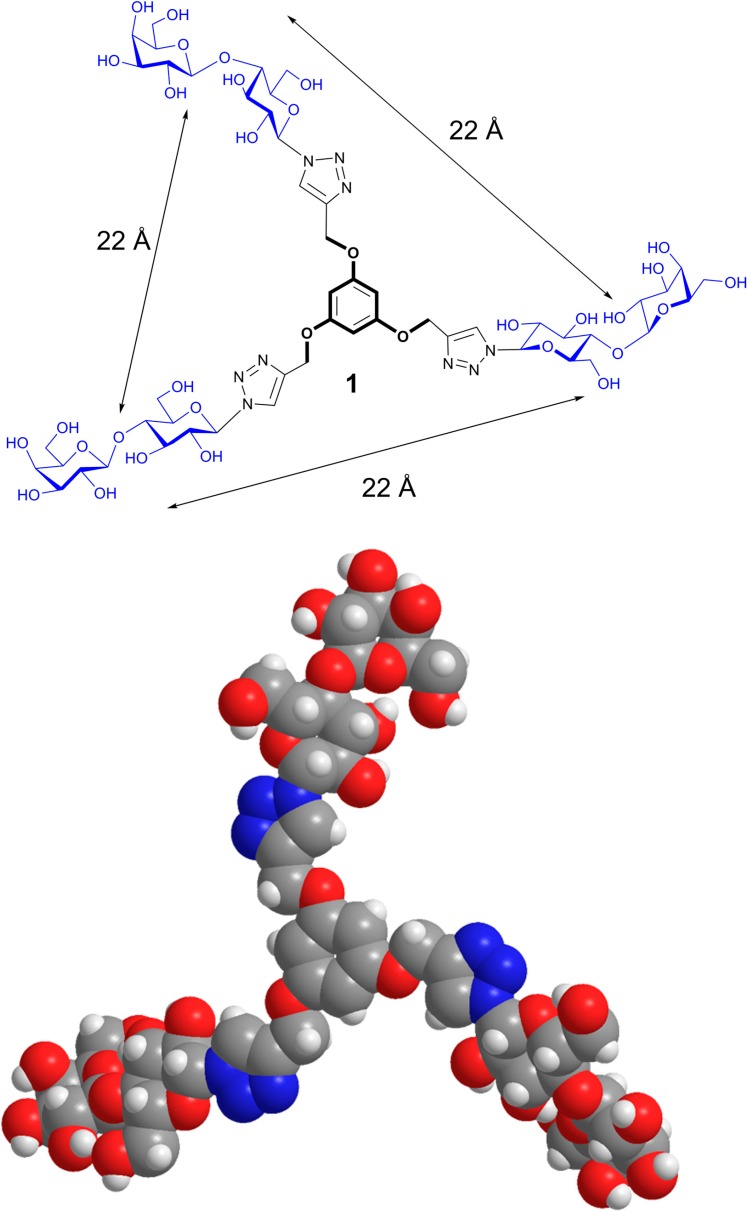
Computer-based estimation of the distances between branch ends of the 1,3,5-trimethoxybenzene-based trivalent glycocluster **1** (for synthetic route, please see Scheme 1) and presentation of the conformer with C-3 symmetry. Dynamic fluctuations in space and time enable the glycocluster to access conformers with distances between 20–25 Å, then losing the C-3 symmetry.

**Figure 13:**
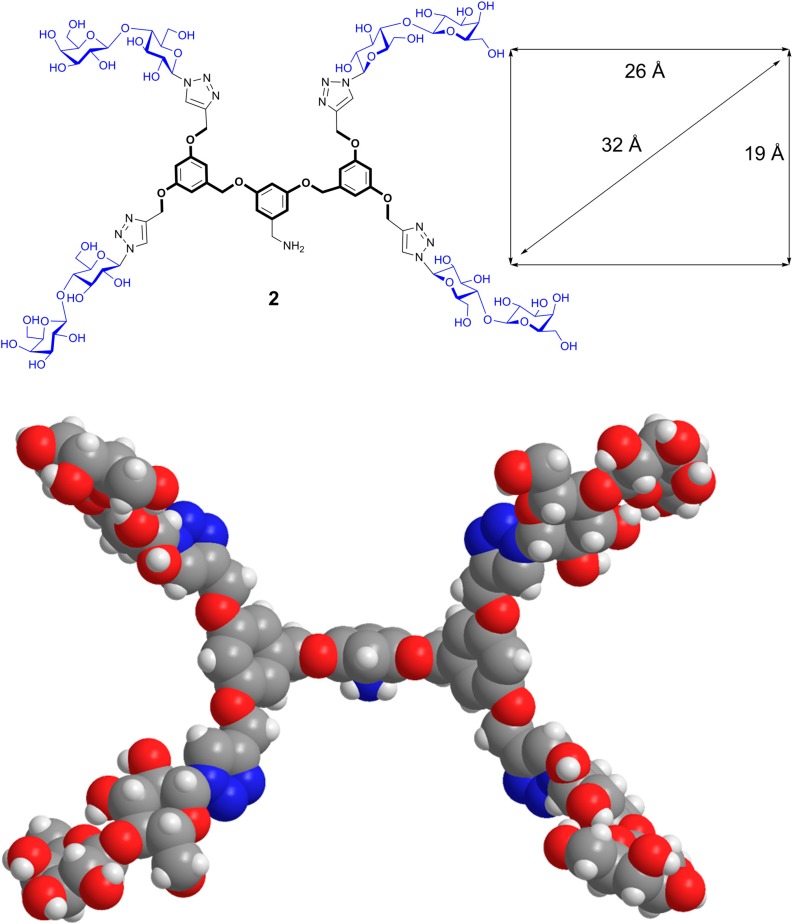
Computer-based estimation of the distance profile between branch ends of the tetravalent glycocluster **2** based on a 1,3-bis(3,5-dimethoxybenzyloxy)benzene core (for synthetic route, please see Scheme 1). The rectangular arrangement accounts for approximate distances of 19, 26 and 32 Å. For a conformer with tetrahedral arrangement of branch ends, please see Figure 14.

**Figure 14:**
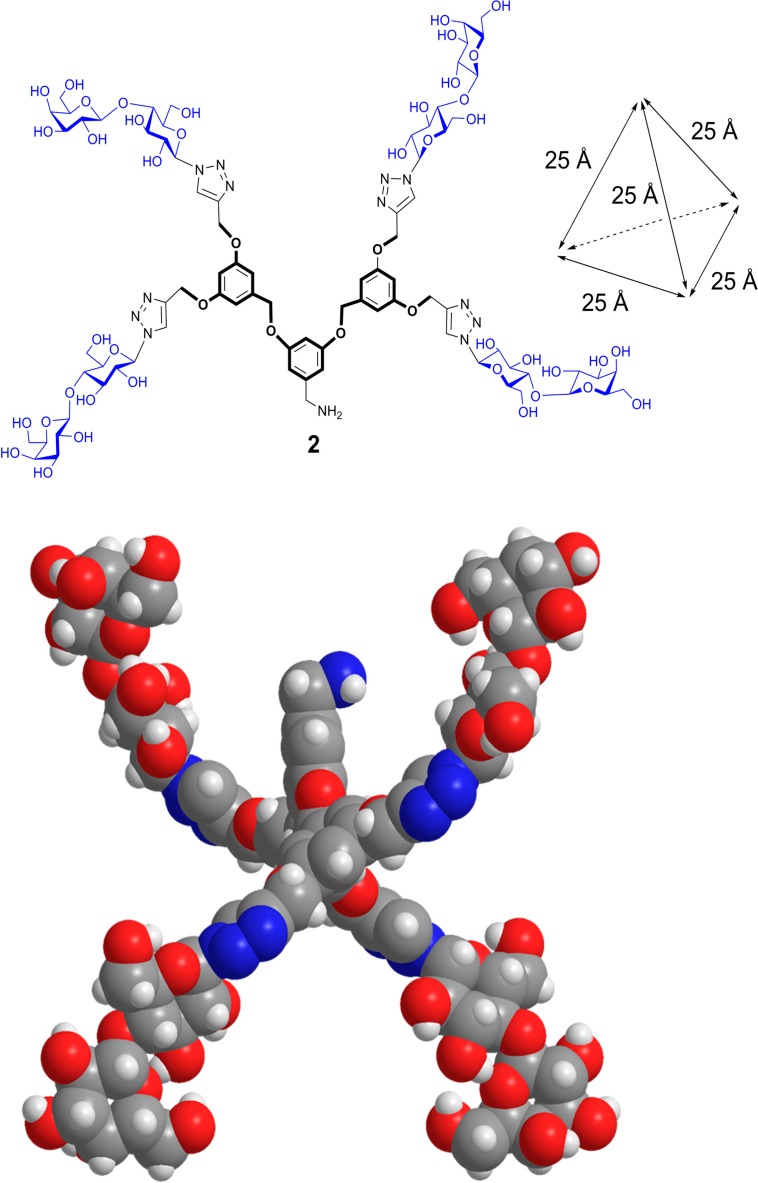
Distance profile of a conformer of the tetravalent glycocluster **2** with tetrahedral branch arrangement. As noted above for the trivalent compound, dynamic fluctuations in space and time will alter the distance profile to a limited extent.

**Scheme 2:**
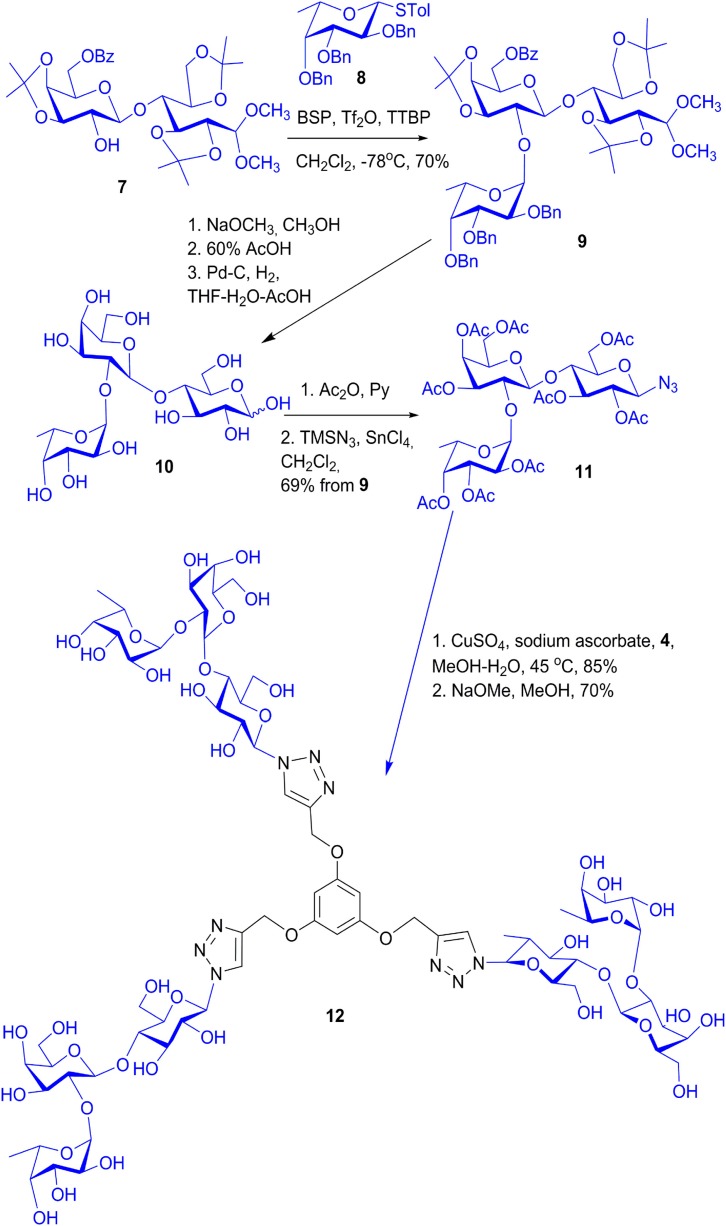
Synthetic route to the trivalent glycocluster with the histo-blood group H(0)-like trisaccharide headgroups (12). Following introduction of the fucosyl moiety using donor 8 into the appropriately protected acceptor 7 and the conversion to the azide 11, the copper-promoted coupling with the alkyne 4 produced the trivalent glycocluster 12 with its trisaccharide (for experimental details, please see [131]).

The intention for the design of scaffolds with several attachment points for carbohydrates is to mimic the branched display of N- and O-glycans. Having identified spatial vicinity as a regulator of lectin affinity, e.g., on level 4, the synthetic work can provide tools for delineating structure-activity profiles. In this sense, placement of sugar headgroups may even access non-physiological constellations, in efforts to optimize affinity and/or selectivity. To qualify as core, the prerequisites of solubility and lack of toxicity must be satisfied, and conjugation of sugars should be possible in a straightforward manner, as is often the case [31,130]. To offer a graphic example, Scheme 1 documents the steps to a trivalent glycocluster starting from 1,3,5-trihydroxybenzene (**1**) using copper-promoted azide-alkyne cycloaddition, and a tetravalent scaffold was produced by the same pathway (for details, please see [131]). Their spatial features of ligand presentation were then characterized by molecular modeling. Resulting low-energy conformers with the average distances between the sugars in the different branches are shown in Figure 12, Figure 13 and Figure 14. These intramolecular parameters in the artificial glycoconjugates lie in the range of what is for example characteristic for a complex-type triantennary N-glycan, as shown above in Figure 3b, a high-affinity ligand for the hepatic asialoglycoprotein receptor [49]. With respect to galectins, their association to each branch end of the bi- and triantennary N-glycans of the glycoprotein asialofetuin has been ascertained by precipitation and calorimetry [132,133]. To test whether this design enhances the bioactivity of the artificial glycoconjugates, assays monitoring lectin binding to a glycoprotein and to cells were performed in the absence and presence of the synthetic compound, measuring extent of signal reduction [31]. The synthetic glycoclusters could block the lectin to a certain extent. The tetravalent compound was more potent as inhibitor for Gal-3 than for proto-type galectins, a result that underlines the impact of topological presentation [131]. A further increase in activity and selectivity of this glycocluster was achieved by headgroup tailoring, extending the disaccharide lactose to the H(0) trisaccharide (Scheme 2) [131].

Inversely, tailoring protein features-while keeping the glycocluster design constant-will raise evidence for functional structure-activity implications from the side of the protein. A pertinent question, to illustrate practical aspects, is what presence and length of the linker in tandem-repeat-type galectins mean for the protein. Engineering on the cDNA level is a way of generating new non-physiological variants of the natural proteins for comparative testing. Shortening the 42-amino-acid-long linker of human galectin-4 (Gal-4) reduced the inhibitory activity of a bivalent 1,4-diethynyl-benzene-based compound more than 20fold [134]. This result is in line with a rather flexible linker, which allows the two CRDs to adapt to slightly different topologies of glycan presentation. This way, two types of counterreceptors can be brought together, e.g., in Gal-4-mediated apical or axonal transport [135,136].

Thus, spatial characteristics on both sides (*i.e.*, glycan and lectin) alter reactivity and lead to an optimal fit, in *cis*- and in *trans*-interactions. This mutually matching orientation is the basis of the lectin-dependent cell aggregation shown in Figure 11. Because this process, also cell adhesion to a matrix as well as initiation of signaling all depend on cell surface binding, tailoring a substratum (mono- or bilayers) or liposomes, also termed nanoparticles, accordingly affords access to respective platforms for the next steps of testing, that is to build models for levels 5 and 6 of affinity regulation. Natural or synthetic glycolipids, together with cholesterol and glycerophosphatides, can be chosen to mimic membrane properties, even at array format [137], and this increasingly so in an iterative series. Self-assembling amphiphilic Janus dendrimers are another starting set for programmable vesicles of a size of choice [138,139]. While studies on a monolayer surface will provide insights into binding and reorganization of monolayer constituents (*cis*-interactions), monitoring aggregation of liposomes or glycodendrimersomes characterizes *trans*-interactions. Admittedly, refining the models to come into the vicinity of the natural complexity of cell membranes will take quite a series of steps. Toward this end, teaming up synthetic and supramolecular chemistry with protein biochemistry will be instrumental. This strategy will help us understand the intriguing counterreceptor specificity by simulating natural systems and purposefully testing parameter changes.

## 7. Conclusions

The intricacies of cell sociology depend on the presentation of a multitude of signals with distinct information content on the cell surface. Because the available space is limited, the structural platform must make high-density coding possible. In chemical terms, more than the sequence is then required when synthesizing oligomers. Carbohydrates fulfill this prerequisite to its full extent, and the ubiquitous presence of glycans (as part of glycoconjugates or as saccharides of variable chain length) led N. Sharon to boldly state that “we know that the specificity of many natural polymers is written in terms of sugar residues, not of amino acids or nucleotides”, although the excitement raised by the insights into glycan diversity “should not hide the fact” that we are just beginning to understand the rules of the sugar code [140]. Determinants of this third alphabet of life offer contact points for interactions, and they can be used by endogenous receptors (lectins). Their presence spread over more than 12 families in mammals stimulates research activities to map characteristics of each lectin thoroughly. The phylogenetic divergence within the lectin families and the development of individually regulated expression patterns, with the possibility for co-regulation in counterreceptor presentation, are strong arguments for the assumedly productive lectin-glycan recognition. To answer the question on how its intriguing specificity is achieved, that is how the right partners are brought together to turn sugar coding into cellular activities, is a demanding challenge. Merging the sophistication of synthetic and supramolecular chemistry with biochemical and cell biological approaches to continue to dissect sugar coding by glycoconjugates and cell surfaces in detail is the methodological answer. This gives a reason to substitute the term “glycobiology” by “glycosciences”, which reflects the interdisciplinarity of the field.
